# Effects of Heterogeneous and Clustered Contact Patterns on Infectious Disease Dynamics

**DOI:** 10.1371/journal.pcbi.1002042

**Published:** 2011-06-02

**Authors:** Erik M. Volz, Joel C. Miller, Alison Galvani, Lauren Ancel Meyers

**Affiliations:** 1 Department of Epidemiology, University of Michigan, Ann Arbor, Michigan, United States of America; 2 Department of Epidemiology, Harvard University, Cambridge, Massachusetts, United States of America; 3 Fogarty International Center, National Institutes of Health, Washington, D.C., United States of America; 4 Department of Epidemiology, Yale University, New Haven, Connecticut, United States of America; 5 Santa Fe Institute, Santa Fe, New Mexico, United States of America; 6 Section of Integrative Biology, The University of Texas, Austin, Texas, United States of America; University of New South Wales, Australia

## Abstract

The spread of infectious diseases fundamentally depends on the pattern of contacts between individuals. Although studies of contact networks have shown that heterogeneity in the number of contacts and the duration of contacts can have far-reaching epidemiological consequences, models often assume that contacts are chosen at random and thereby ignore the sociological, temporal and/or spatial clustering of contacts. Here we investigate the simultaneous effects of heterogeneous and clustered contact patterns on epidemic dynamics. To model population structure, we generalize the configuration model which has a tunable degree distribution (number of contacts per node) and level of clustering (number of three cliques). To model epidemic dynamics for this class of random graph, we derive a tractable, low-dimensional system of ordinary differential equations that accounts for the effects of network structure on the course of the epidemic. We find that the interaction between clustering and the degree distribution is complex. Clustering always slows an epidemic, but simultaneously increasing clustering and the variance of the degree distribution can increase final epidemic size. We also show that bond percolation-based approximations can be highly biased if one incorrectly assumes that infectious periods are homogeneous, and the magnitude of this bias increases with the amount of clustering in the network. We apply this approach to model the high clustering of contacts within households, using contact parameters estimated from survey data of social interactions, and we identify conditions under which network models that do not account for household structure will be biased.

## Author Summary

The transmission dynamics of infectious diseases are sensitive to the patterns of interactions among susceptible and infectious individuals. Human social contacts are known to be highly heterogeneous (the number of social contacts ranges from few to very many) and to be highly clustered (the social contacts of a single individual tend also to contact each other). To predict the impacts of these patterns on infectious disease transmission, epidemiologists have begun to use random network models, in which nodes represent susceptible, infectious, or recovered individuals and links represent contacts sufficient for disease transmission. This paper introduces a versatile mathematical model that takes both heterogeneous connectivity and clustering into account and uses it to quantify the relative impact of clustered contacts on epidemics and the prediction biases that can arise when clustering and variability in infectious periods are ignored.

## Introduction

Contacts sufficient for transmission of infectious disease occur repeatedly within stable relationships such as between sex partners or within households and workplaces. Epidemiologists increasingly use random network models that explicitly capture such interactions to study disease dynamics [1]. This work has shown that infectious disease dynamics can be profoundly influenced by two key network properties– the distribution in the number of contacts per individual (the degree distribution) [2] and the transitivity or clustering of contacts, such as within households [3], [4]. However, we lack a general framework for studying the combined epidemiological impacts of clustering and degree distribution. For public health, such understanding may be critical to predicting epidemiological events across diverse populations and tailoring control strategies appropriately.

As epidemiological models grow in complexity, we face the question of how much complexity is necessary and useful. For example, which features of network structure significantly influence disease dynamics and which can we ignore without introducing large biases? In some cases, mass action models that assume panmixis may be adequate and thus we can ignore network structure altogether. In others, incorporating realistic degree distributions and/or clustering may be important. A published simulation-based study [5] suggests that clustering affects epidemic dynamics when transmissibility is low and contacts between two individuals are highly autocorrelated. However, there remains a clear need for general, systematic model selection rules.

The impact of the degree distribution on epidemics in the absence of clustering is complex, but has received considerable attention and is relatively well understood [1], [2], [6], [7]. For example, in networks with power law degree distributions (so-called scale free networks), as the variance of the degree distribution diverges to infinity, the reproduction number for a given pathogen also diverges to infinity while the minimum transmissibility necessary for epidemics to occur approaches zero (meaning even diseases with very low infectiousness have the potential to cause epidemics).

In contrast, the effects of clustering on epidemics are still unclear. Some studies suggest that clustering decreases epidemic thresholds, making an epidemic more likely to occur after an initial introduction [8]. Others studies suggest that the relationships between clustering and the epidemic threshold is subtle [9]–[11], and depends on the nature of clustering in the population. The effects of clustering on the timescale of an epidemic are less ambiguous, with most studies suggesting that clustering decreases the rate of epidemic propagation. Here, we describe and analyze a versatile model that allows extensive exploration of the interactive impacts of clustering and degree distribution on epidemic dynamics. Although clustering always retards an epidemic, the timescale of the epidemic is more sensitive to the variance of the degree distribution than to clustering.

Following the approach introduced in [12], [13], we model the spread of infectious disease through structured host populations using networks that are straightforward generalizations of the configuration model [14]. Our model is designed so that one can easily tune the parameters describing the degree distribution and the number of cliques in the network (a *clique* is a completely connected subgraph), which is closely related to the clustering coefficient. Although these networks are not tree-like locally, they can be analyzed using branching processes and percolation theory, as shown in [12], [13], and more recently in [15] and [16].

Our epidemic model generalizes the approaches recently introduced in [17], [18] for modeling the dynamics of epidemics in networks. These models exactly predict epidemic spread in a class of random networks. The resulting model consists of a low dimensional system of ordinary differential equations that describes the prevalence of infection over time. Recently, an alternative system of approximate ODEs was independently developed [19] which describes epidemics in networks with arbitrary degree distributions and clustering coefficients. This heuristic approach is intended to be fairly generic, and it is not clear if there are clustered networks for which this model is exact. Our complimentary approach allows straightforward analytical solutions (using percolation theory and branching process methods) for a simple class of random networks. In some cases, our model agrees closely with the one presented in [19], but it can differ substantially around epidemic thresholds. This result suggests that the clustering coefficient (a single value for the entire network) alone is not always sufficient to determine the full epidemiological impact of clustering.

We also revisit one of the early, pioneering approaches to modeling disease transmission through complex contact networks: approximating the final size of an epidemic (the giant component of the network) using bond percolation [12], [13]. A recent paper introduces a method that correctly accounts for variation in infectious periods when making such calculations [16]. In contrast to what is found in unclustered networks, in which such variation does not significantly impact epidemic sizes [20]–[23], we find that in highly clustered networks ignoring variation in infectious periods can introduce considerable bias.

In addition, we model a realistic population by estimating network parameters from a large diary-based survey of social interactions [24]. We quantify the amount of network clustering that occurs within households and show that ignoring household clustering can lead to significant prediction errors including overestimation of both prevalence and, somewhat counter-intuitively, the epidemiological significance of households.

## Materials and Methods

We consider a basic susceptible-infected-recovered model. Infectious nodes transmit to neighbors at a constant rate 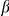 and transition to the immune recovered state at a constant rate 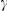. Once recovered, the node cannot be re-infected, and can no longer transmit to neighbors. Key parameters and variables are defined in table 1.

**Table 1:** Definitions for key parameters and variables.

Parameter	Definition
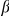	Transmission rate
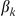	Transmission rate within a clique of size 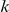
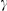	Recovery rate
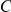	Clustering coefficient
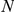	The number of nodes in the network
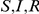	The fraction of the population susceptible, infectious, and recovered respectively
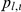	The frequency of nodes in the network that is a member of 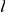 lines and 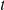 triangles
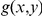	Probability generation function for the numbers of lines and triangles of which a node is a member
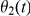	A survivor function for remaining susceptible given that a node is a member of a single line
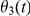	A survivor function for remaining susceptible given that a node is a member of a single triangle
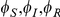	The probabilities that a neighbor of a susceptible node along a line is susceptible, infectious or recovered
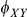	The probabilities that the two neighbors of a susceptible in a triangle are in states 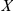 and 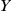
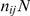	The number of 3-cliques with 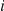 susceptible and 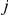 infectious members
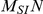	The number of lines with one susceptible and one infectious member

Our solutions are based on the class of undirected random graphs originally described in [12], [13], which are refinements of bipartite configuration models [8], [25], [26]. A node can be a member of multiple cliques of various size. A two-clique is a pair of nodes with an edge between them, and we will call these *lines*. A three-clique is three nodes with all three possible edges, which we call *triangles*. Each node is a member of a random number of lines and triangles. The probability that a node is a member of 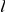 lines and 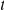 triangles is described by the probability mass function 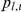. Our model captures network structure using the probability generating function (PGF):
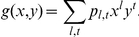
The degree distribution, which describes the probability that a node is a member of 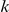 edges, is generated by the following univariate PGF:
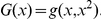
Finite-size realizations of these random networks can be easily generated as described in the next section. Most of this section concerns the derivation of equations that describe epidemic dynamics; these solutions are asymptotically exact in the limit of large population size, and as discussed below, compare well to large random networks.

Clustering is often characterized using the clustering coefficient, 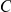, which is the ratio of 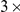 the number of triangles [12], [13], denoted 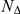, to the number of 2-paths in the network, denoted 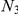. 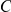 can be interpreted as the probability that two random edges that share a common node are joined by a third edge to form a triangle. Thus we have
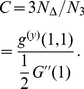
(1)


When differentiating the PGF, we will use superscripts so that, for example, 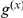 would indicate the first derivative with respect to 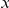 and 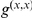 would indicate the second derivative with respect to 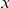. The PGF can be used to calculate many useful properties of the graph; for example, the expected number of lines and triangles to which a random node belongs is
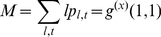
(2)

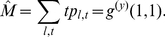
(3)


### Generating random clustered networks

Random graphs [12], [13] can be algorithmically generated by assigning a random number of lines and triangles to a set of 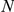 nodes from the distribution 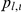. Edges can then be created by

1. generating a set of half-lines or “stubs”, such that the number of times a node appears in the set is equal to the number of lines to which it belongs,
2. generating a set of “corners”, such that the number of times a node appears in the set is equal to the number of triangles to which it belongs,
3. ensuring that the number of stubs is divisible by two and the number of corners is divisible by three, for example by randomly deleting any remainder,
4. repeatedly constructing an edge between two stubs drawn at random and without replacement,
5. and, repeatedly constructing edges between three corners drawn at random and without replacement.

This algorithm may produce loops and double-edges, but the frequency of such edges will be negligibly small for large graphs [27], and we simply delete them if they do occur.

### Disease transmission through clustered random networks

The ODEs that describe epidemic dynamics in clustered networks can be expressed in several equivalent forms and derived from at least two different perspectives. Below, we present two systems of equations that respectively describe the change in the number of cliques with 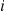 susceptible and 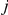 infectious nodes and the probability that a susceptible node is connected to such a clique. Both of these systems can also describe the dynamics of the number of infected and susceptible individuals in the population as a function of time. First we present the system of equations based on the probabilities 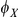 that a random node 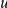 is connected by a line to a node in state 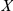 and the probabilities 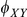 that 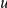 is connected in a triangle to two nodes in states 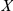 and 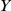. Below we present an alternative derivation based on the numbers of cliques with different configurations. The derivation of this system is very similar to what was presented in [28], but is less mathematically parsimonious than the system of equations in this section, which requires only 7 ODEs. And, below we show how this system can be extended to networks with generalized distributions of clique sizes, that is, networks that include cliques larger than size three.

We follow the recently introduced edge-based compartmental modeling approach of [18]. This approach is based on the consideration of the fate of a single randomly chosen node 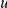 in the network. The probability this node is susceptible is equal to the proportion of nodes that are susceptible, and the probability it is infected or recovered is similarly the proportion of nodes that are infected or recovered. If we know the probability the node is susceptible as a function of time, then we can calculate its probability of being infected or recovered, so we focus our attention on calculating 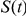, the probability the randomly chosen test node is susceptible. Following [18] we modify the test node so that it does not transmit infection once infected. This does not alter the probability it is susceptible, but eliminates some conditional probability arguments we would have to consider otherwise.

Assume 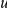 is a member of 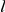 lines and 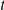 triangles. Then the probability it is susceptible is 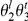 where 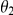 is the probability that a random line has not transmitted to the test node and 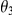 is the probability that neither of the other nodes in a triangle has transmitted to the test node. So assuming we can calculate 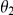 and 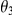 as functions of time, we have 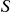 as a function of time. From this we use 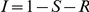 and 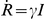 to find 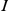 and 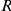.

**Figure 1:**
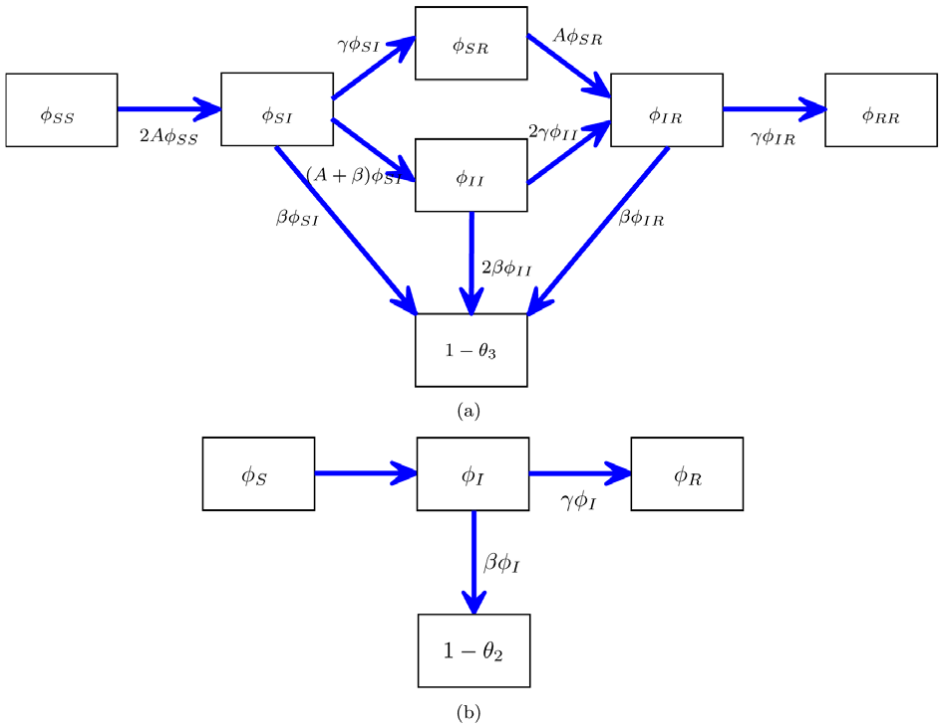
A schematic of the system of equations 7–8. A: The flux between the probabilities that a node 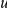 is connected to a triangle with all possible configurations as well as the probability that a node 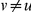 in the triangle has transmitted to 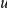. B: The flux between the probabilities that a node 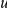 is connected by a line to a node 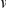 that is susceptible, infectious, recovered, and the probability that 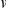 has transmitted to 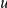.

Let us first consider 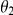. We divide 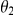 into 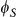, 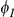, and 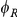, the probabilities that a neighbor along a line has not transmitted infection to 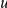 and is either susceptible, infected, or recovered respectively. The probability the neighbor has not transmitted is
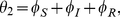
(4)and 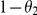 is the probability that it has transmitted. We create compartments for these states and display the flux between them in Figure 1.

The fluxes from 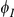 to 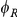 and 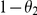 are proportional to each other, and each begins as zero, so we can show that 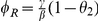. We find 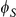 by a different approach, similar to the calculation of 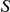. A neighbor found along a randomly chosen line will tend to have more lines than a node chosen uniformly at random. The random number of such lines is described by the *excess degree distribution* [29], and we calculate the generating function for this distribution as follows. Denote 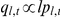 to be the probability that there are 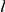 lines and 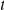 triangles connected to a susceptible node that we reach by following a line from an infectious to a susceptible node not counting the line by which we arrived. Similarly, 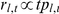 is the probability that if we follow a triangle to a susceptible node, there are 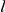 lines and 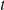 triangles connected to that node, not counting the one by which we arrived. Then we have the generating functions
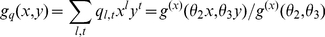
(5)

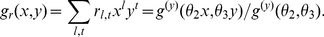
(6)Equations 5 and 6 generate the excess degree distributions for lines and triangles.

A neighbor reached by following a line connected to 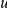 is susceptible with probability 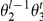 (recall that 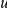 does not cause infection) where 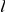 is a realization of the excess degree distribution. Summing over values of 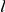, we find 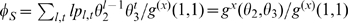. Now we rearrange equation 4 which gives 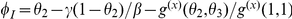.

We can finally calculate 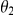 by noting that Figure 1 shows 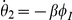. We find
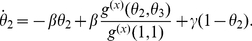
(7)


To complete the system, we need a corresponding equation for 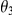. Here the system is more complicated. For the line case, if the neighbor had not transmitted, there were just three states to consider. But when considering triangles, if neither neighbor has transmitted, there are 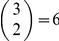 states to consider. We define 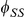 to be the probability both neighbors are susceptible, 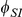 to be the probability one neighbor is susceptible, while the other is infected but has not transmitted to 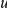, 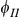 to be the probability both are infected but neither has transmitted to 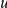, and similarly define 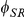, 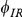, and 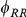. Figure 1 shows the compartments and flux between them.

We do not have a simple relation for 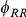 and 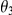, so our derivation changes mildly. The starting point will be 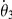, which satisfies
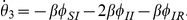



To calculate the right hand side, we first find 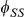, the probability that both neighbors in a triangle are still susceptible. Under the assumption that transmissions have not happened in the triangle, the probability that one neighbor is still susceptible is 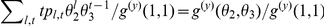. Since we require both be susceptible,
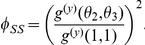
We take 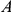 to be the rate that a neighbor in a triangle is infected from outside the triangle. Then 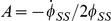. After some simplification, we find
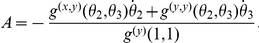
We are now ready to find equations for 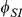, 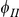 and 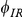. We will also need to find 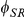 to complete the system, but we will not need 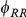. We find
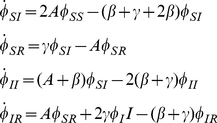
(8)


This completes our system of equations. We are able to calculate 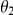 and 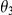 as functions of time, which in turn leads to 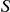, from which we can find 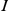 and 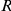 as well:
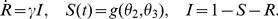
(9)


#### Alternative derivation of epidemic dynamics

This model is based on the idea that the number of transmissions events in the network per unit time is a linear function of several time dependent variables:

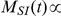
 the number of lines that begin at a susceptible node and terminate at an infectious node,

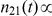
 the number of triangles with two susceptible nodes and one infectious node,

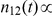
 the number of triangles with one susceptible and two infectious nodes, and

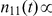
 the number of triangles with one susceptible node, one infectious node, and one recovered node.

The variables 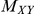 are dimensionless quantities that do not depend on 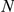. For comparison to simulations, the number of half-lines 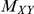 would be 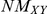. The constant of proportionality depends on the variable under consideration. Given a graph size 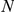, the total number of lines and triangles in the graph are respectively
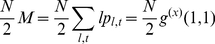
(10)

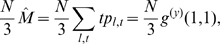
(11)since there are 2 nodes per line and 3 per triangle. For the variables 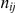 defined above, the total number of triangles is 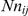. And the total number of lines between susceptibles and infected is 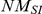. However, below we also use the variable 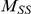 which is proportional to the number of lines connecting two susceptibles. In this case, the total number of such lines is 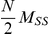 since this variable counts lines twice (once for each susceptible node in the clique).

We will assume that the number of transmissions per unit time over a line or triangle are proportional to
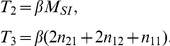
To model epidemic spread, we construct a set of ODEs in terms of the 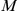 and 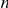 variables as well as two survivor functions for susceptible nodes [17], [30]:

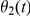
: the probability that a neighbor in a “line” has not transmitted infection prior to time 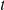, and

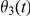
: the probability that both neighbors in a “triangle” have not transmitted infection prior to time 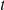.

The probability that a node with 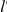 lines and 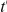 triangles remains susceptible is 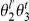 (see [17], [30] for a justification). Consequently the fraction of the population, 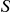, that remains susceptible at any time is
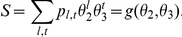



The probability that an edge beginning at a susceptible node will terminate at an infectious node is 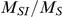, where 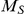 is proportional to the number of half-lines or *stubs* connected to susceptible nodes. Similarly, the probability that a susceptible node is connected to a triangle with 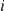 susceptible nodes and 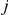 infectious nodes is 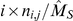.These two variables can be expressed in terms of the PGF:
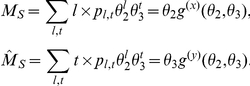



The system of ODEs relies on several more variables derived from the generating function. When a transmission event occurs, lines and triangles that were formally counted among 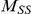 or 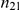 may instead be counted among 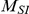 or 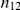. Quantifying the magnitude of these changes requires that we calculate the average degree of a newly infected node. This is accomplished with the excess degree distribution and its corresponding generating function [29] (equations 5,6). The mean number of lines and triangles in these joint distributions gives us the expected number of lines or triangles of a newly infected node. We denote the means as 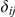, which is the average excess number of type-j links for a susceptible node selected with probability proportional to the number of type-i links. Using the generating functions, we have
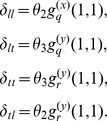
(12)


The hazard of infection along a single edge is proportional to the probability that the edge terminates at an infectious node (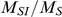) and the transmission rate, implying [17]

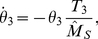
(13)

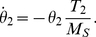
(14)


Dynamics of 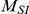 and 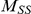 require careful consideration of how edges are rearranged following a transmission event. 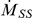 describes the time derivative of the normalized number of lines between susceptibles. 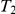 transmissions occur per unit time along lines, and the newly infected individual is connected to an average of 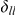 lines in addition to the one by which the individual was infected. The probability that such a line is shared with a susceptible node is the ratio of the number of lines between susceptibles to the total number of half-lines connected to susceptibles: 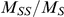. Note that this probability does not correspond to what we would have in randomly mixing population, which would just be the fraction of susceptible half-lines in the network: 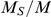. The extent to which 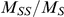 differs from 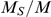 reflects the extent to which the state of neighbors in the network is correlated due to the spread of the epidemic. Therefore, 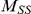 will decrease at a rate of 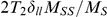.

Furthermore, 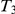 transmissions will occur via triangles, and the newly infected node will be connected to an expected number 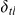 lines. Each of these will also terminate at a susceptible node with probability 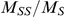. Then we conclude
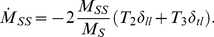
(15)


The equation for 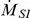 can be derived similarly. The edge rearrangement follows a similar pattern as for 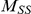, but we must account for the increase of 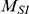 when a newly infected node is connected to another susceptible (with probability 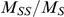) and the decrease of 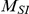 when the new infection has connections to other infecteds (with probability 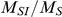). Then the new infection has connections to other infecteds (with probability 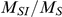), yielding terms of the form 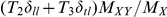.

In addition to the edge-rearrangement terms, we must account for changes due to recovery (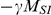) and direct transmission (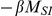).
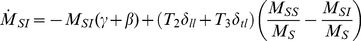
(16)


Finally, the equations for the number of triangles with 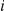 susceptible and 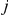 infectious constituents, 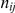, is found by considering rearrangements as above, as well as flux between classes that are due to an infectious member of the triangle transmitting to a susceptible member, or recovering. For example, a triangle with one susceptible and two infectious nodes (state 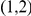) will transition to the state 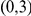 at the rate 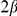, because there are two edges between susceptible and infecteds in this clique. It will also transition to the state 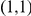 at the rate 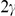, because there are two infectious nodes in the clique that can recover. To summarize, we find
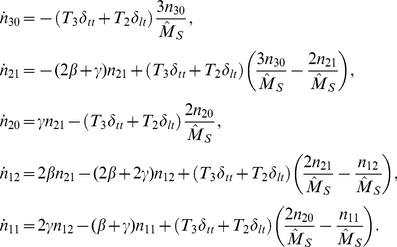
(17)


An extra differential equation can be solved for the epidemic prevalence at any time.
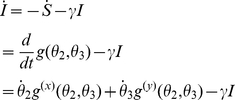
(18)This system can also be related to the one in the previous section by the change of variables 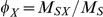 and 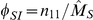, etc.

If an initial fraction 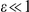 of the population is infected at the beginning of the epidemic and the total number of lines and cliques are respectively proportional to 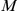 and 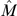 (equation 3), we use the initial conditions
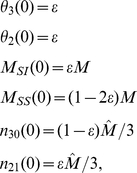
(19)and the remaining variables would be zero.

### Generalization to clique sizes 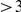


It is straightforward to generalize the derivation for triangles (3-cliques) to larger clique sizes, and to furthermore allow the transmission rate to be a function of clique size. Let 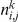 denote the number of cliques of size 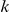 with 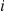 susceptible and 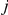 infectious nodes. We will generalize the preceding model to allow transmission rates to vary between cliques of different sizes. The transmission rate for edges within a clique of size 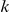 will be denoted 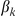. We consider clique sizes from 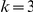 to a maximum of 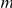. Having multiple clique sizes requires us to introduce additional dummy variables into the generating function. The vector 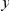 of dummy variables with elements 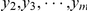 correspond to each of the 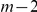 clique sizes and unclustered edges. Note that the element 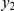 is the dummy variable corresponding to lines, previously denoted 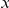. Then the following will generate the degree distribution:
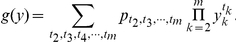


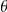
 will be the vector of survivor functions with elements 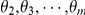.

Letting the derivative of 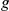 with respect to the dummy variable 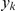 be denoted 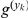, the number of cliques of size 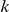 in the network is proportional to 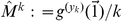 (because there are 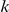 nodes for every 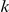 clique). In addition, the number of links from susceptible nodes to cliques of size 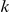 is 
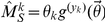
.

We find the dynamics of 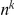 by tabulating the flux to and from cliques with similar configurations. A 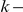
clique with 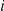 susceptible and 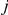 infectious nodes will have 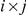 edges between susceptible and infectious nodes, so that transmissions within cliques will occur at the rate 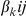. The rate of transmissions that occur within cliques of size 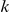 is
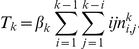
The rate of transmissions by unclustered edges will be 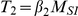, and nodes in cliques of size 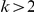 with 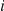 susceptible and 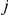 infectious nodes will be infected from outside of the clique (i.e. by an edge with an infectious node not in the clique) at a rate
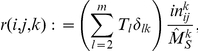
where 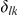 is the average number of 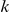 cliques of a node selected by randomly choosing a susceptible member of a random 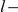
clique:
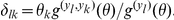
A clique with 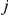 infectious nodes will have recovery events at the rate 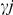.

Putting these terms together yields the following solution for the dynamics of 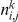. These equations are defined for all 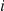 and 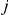 such that 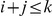.
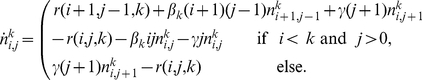
(20)


The survivor functions will be determined by the following set of differential equations:
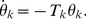
(21)


The equations for 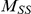 and 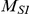 will be the same as equations 16 and 16, except that indirect transmissions by cliques larger than three must be taken into account.
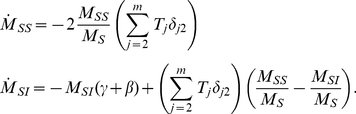
(22)


Calculation of the survivor functions only requires cliques such that 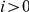 and 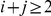, so it is not necessary to solve for all possible configurations of 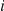 susceptible and 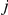 infectious nodes. In general, if cliques range in size from 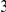 to 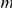, this will require 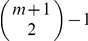 equations.

### Bond percolation approximations for final epidemic size

For an infectious disease spreading in a population in which all individuals have the same susceptibility and the same infectiousness and all transmissions are independent, the epidemic process can be exactly represented through a bond percolation process. Consider an individual 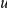 chosen to be the initial infection. Assume the per-contact probability of transmission is 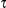. If we delete each edge of the network with probability 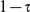, then the probability that 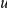 is in the same component of the residual network as a given set of nodes is equal to the probability that that set of nodes is infected in the epidemic [20], [23], [31].

However, if there is variable infection duration or some other cause of heterogeneity in infectiousness, this is no longer the case: those individuals with longer infectious period are more infectious. Assuming the only heterogeneities are due to variable infectiousness, it has been shown [22] that in networks without short cycles the final size of large outbreaks depends only on the average infectiousness in the limit of large networks.

When there are short cycles, the size of epidemics does depend on how infectiousness is distributed. The assumption that all individuals have the average infectiousness only gives an upper bound on epidemic size [23], [31]. This bound is often a reasonable approximation [9]. Recently, an alternative percolation technique was developed [16] which accounts for variable infectious periods and can accurately calculate final sizes in some clustered networks.

Taking the transmission rate to be 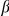 and the recovery rate to be 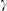, the average probability of infecting a neighbor is 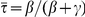. First, we investigate how closely the bond percolation approach reproduces epidemics with constant transmission and recovery rates for the clustered networks considered here. Second, we present an alternative simple solution for final size in clustered networks that takes variable infectious periods into account.

The original bond percolation method for clustered networks [12], [13] can be used to determine the probability that there would be zero, one or two secondary infections following an initial infection in a triangle. If the transmission probability 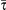 is constant, the probability of having one or two secondary infections in a triangle is (refer to [12], [13]):

- one secondary infection: 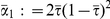,
- two secondary infections: 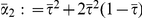.

In fact, these probabilities are functions of the infectious period of the initial case in a triangle, which is itself an exponentially distributed random variable. We can solve for the true probabilities by integrating over the infectious period (in this case 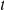 denotes time). Conditional on the infectious period being 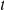, the probability of transmission by single infected to a single neighbor of that infected is 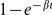. When the infectious period is exponentially distributed with rate 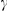, we have the following:

- One secondary infection:
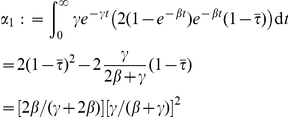

- Two secondary infections:
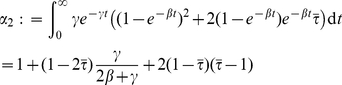

This distribution is generally different from the one based on 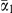 and 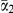, and the expected number of secondary infections is strictly less with variable infectious periods. To see this, we denote the averages 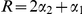 and 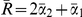, and note that only second order terms of 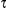 will differ between 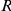 and 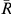. We have 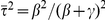, and
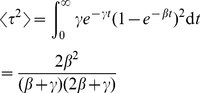
(23)It is straightforward to see that 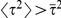. Furthermore, if we collect all terms involving 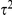 in the equation for 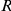, we find a leading factor of 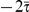. Consequently, these terms will be negative and will have larger magnitude in the expression for 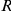 than for 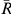, so 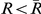.

Now we present an asymptotically exact solution for final epidemic size. Let 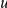 be a random node. Let 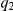 be the probability that a neighbor of 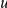 along a line is not infected from another node at the end of the epidemic. Then following the methods described in [12], [13], this probability must satisfy
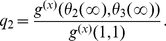
(24)Similarly, let 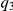 be the probability that a neighbor in a triangle never receives an infectious dose from outside that triangle.
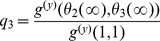
(25)We need to calculate 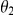 and 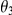 at 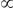 in order to calculate final epidemic size. It suffices to find 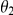 and 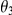 in terms of 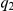 and 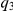 and then solve the system.

We have 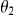 is the probability that a line does not transmit to 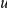. Clearly this can be calculated by considering the probability the neighbor is never infected plus the probability the neighbor is infected, but does not infect 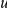. This is
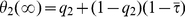
(26)Finding 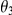 is slightly harder. This is the probability that neither neighbor in a 3-clique is infected from outside, or exactly one receives infection from outside, or both receive infection from outside and transmission does not reach 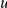. As above, 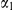 is the probability that a node in a triangle will lead to exactly one further transmission within the triangle, and 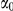 will be the probability it will cause no transmissions. The probability that an infected neighbor in a triangle recovers prior to transmitting to either of its neighbors is 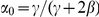. Then
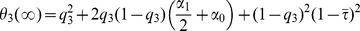
(27)The first term means neither neighbor is infected. The second term has exactly one neighbor infected (factor of 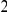 because there are two choices), with the neighbor either infecting the other neighbor, but nothing further or the neighbor infects no one. The third term is both getting infected from outside; we do not need to consider the correlations in this case.

Equations 24–25 can be solved numerically by iteration from small initial values of 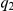 and 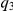 [12], [13]. Given 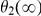 and 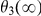, the final size can be calculated:
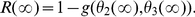
(28)


In the SI, we show how these calculations can be extended to models with generalized distributions of clique sizes.

### Comparison to alternative models

To validate the model assumptions, we compare solutions of the system given by equations 13–17 to stochastic simulations in continuous time based on the Gillespie algorithm [32]. Random networks are generated as described above. At time 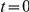, a number of 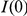 initial infections are selected uniformly at random within the network. When a susceptible is infected, new transmission and recovery events are queued with exponentially distributed waiting times.

We also compare our model to a similar model consisting of ODEs based on moment-closure [19]. This model was developed for networks with a given degree distribution generated by 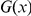 and a clustering coefficient 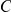. Unlike our model, this system does not specify a joint distribution for the number of lines and triangles. Rather, this system is based on the concept that potential triangles, of which a degree 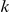 node will have 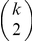, will exist with independent probability 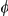. This system also uses PGFs within a low-dimensional system of ODEs, and proposes that 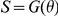, with 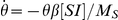, where 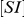 is the number of half-edges from a susceptible node that terminates at an infectious node. Equations for 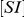 are derived in terms of the number of connected triples, or 2-paths, of nodes that pass through a susceptible. This model makes the approximation that the number of 2-paths connecting two susceptibles and an infected is a simple function of the clustering coefficient 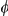:
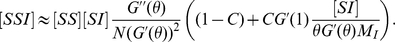
The number of 2-paths connecting a susceptible with two infecteds is
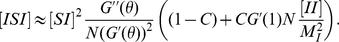
We will subsequently refer to this as the House-Keeling (HK) model.

## Results

We used our low-dimensional model to explore the interactions between the variance of the degree distribution and the level of clustering, as they impact epidemic dynamics. To do this, we constructed a negative binomial degree distribution which allows us to hold the mean degree constant while interpolating variances that range from the mean of the distribution to infinity. The negative binomial distribution with parameters 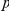 and 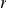 is generated by
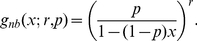
(29)We modified this distribution so that a tuneable fraction 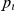 of edges are part of a triangle while keeping the mean of the distribution constant. To construct this distribution, we modify the PGF so that all edges occur in pairs; the degree will always be an even integer. The number of *pairs* of edges follows a negative binomial distribution. With probability 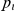, a pair of edges is part of a triangle, and with probability 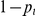, the pair of edges forms two lines with nodes that are not themselves connected. Because lines always appear in pairs, it is easy to keep the mean of the distribution constant while tuning the amount of clustering with 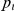, which can range between zero and one. Then given a random number 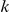 2-tuples generated by equation 29, the number of lines and triangles was generated by 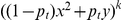, where 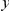 is the dummy variable for triangles, and 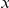 is the dummy variable for lines. Note that the exponent of 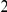 for 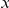 causes all lines to occur in pairs. Using the composition property of PGFs, the degree distribution can be generated by
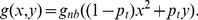
(30)


We compared solutions of the clustering model to 50 stochastic simulations on random networks with 5,000 nodes and 10 initial infections (Figure 1).

The degree distribution was generated by equation 30, with a mean of 2 and a variance of 3. The fraction of edges that are part of a triangle was 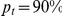. For comparison, we also plot a solution to the clustering model with 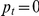, so that there is no clustering. Our results show that clustering slows the epidemic and reduces the final number ultimately infected. The system of equations 13–17 correctly predicts the final size, while the trajectory passes through the central mass of simulated trajectories. The analytical model approximately corresponds to the median time for a stochastic simulation to reach a given prevalence.

**Figure 2:**
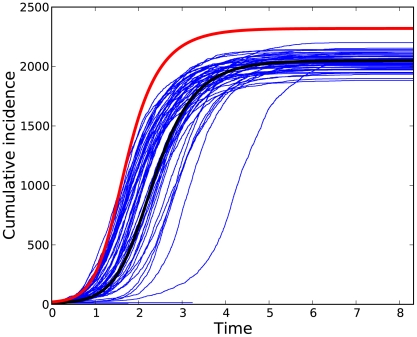
Cumulative number of infections through time. Fifty stochastic simulations (blue dashed lines) are compared to the solution of equations (black line) 13–17. The degree distribution is generated by equation 29 with 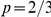 and 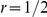. 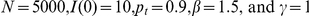. For comparison, a trajectory with 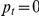 is shown in red.

**Figure 3:**
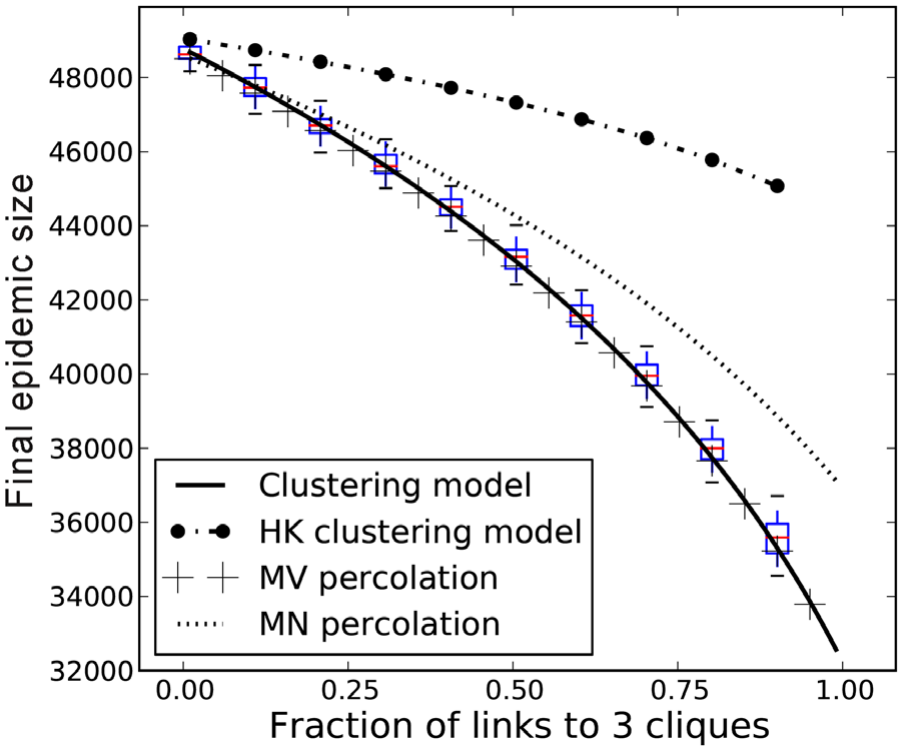
Comparison of clustering models. The degree distribution is Poisson for the number of pairs of edges (mean degree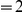). The black line corresponds to the solution of equations 13–17. The boxplots illustrate the 90% confidence interval from 50 stochastic simulations on networks with 5000 nodes. The remaining trajectories correspond to to the original bond percolation calculations [12], [13], our modified bond percolation calculations, and the HK clustering model [19], respectively. 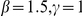.

We examined the effects of clustering on the final size of the epidemic (Figure 2). The clustering model (equations 13–17) correctly reproduces the final epidemic size observed in simulations. However, the MN percolation solution [12], [13] is noticeably biased for non-zero clustering, although it does correctly trend downwards (Figure 3). Over-estimation of the final epidemic size by the MN model is expected because the number of secondary infections within a triangle is overestimated when the infectious period is not constant, as detailed in the methods section.

To calibrate the HK model with our chosen 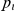, we used the univariate generating function
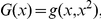
as there are two edges for every triangle. The HK clustering model also overestimates final size for this class of random graph, which is not unexpected, because the HK model assumes a different mechanism for generating transitivity in the network. The lack of alignment between the HK model and equations 13–17 indicates that clustering can impact disease dynamics not only through macroscopic effects such as the clustering coefficient, but also through microscopic characteristics. As we show below, the discrepancy between the HK and clustering models is greatest when the variance of the degree distribution is low; and the large discrepancy between the two models in Figure 3 occurs at the lowest variance considered.

When we systematically explored the effects of the variance of the degree distribution and clustering on the estimated final size of an epidemic, we found that the final epidemic size decreases as clustering increases (Supporting Figure 1 in Text S1). Consistent with previous studies, the final size usually decreases as variance increases. This can happen, for example, if the degree distribution has more nodes with degree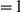 when it is more skewed, which are easily isolated from the giant component. There is an exception, however, when the variance is very small, and clustering is high. In this region, with variance between 1 and 1.5, the final epidemic size can actually increase with larger variance.

We also examined the bias (absolute difference from the true value) of alternative calculations of final size as a function of the variance of the degree distribution and clustering (Supporting Figure 1 in Text S1). The bias of percolation approximations increases with clustering in all cases. However bias is insubstantial when the variance is large, even if clustering is also large. This is a result, at least in part, of the nonlinear relationship between 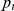 and the clustering coefficient. Given a constant fraction of links to triangles, 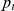, the number of triangles in the network is
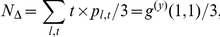
which is constant with respect to the variance of the degree distribution (holding the mean constant). The number of paths with two edges, that is the number of connected triples is
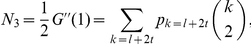
which increases with the second moment of the distribution (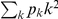). Thus, increasing the variance of the distribution (holding the mean constant) decreases the ratio of 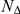 to 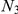. The clustering coefficient, 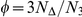 is more important than the total number of triangles in determining epidemic outcomes. As we increase variance, 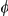 converges to zero, and the clustering model converges to the percolation and HK model solutions. Variance and 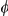, rather than 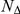, are the important quantities for determining final size, because as the variance of the degree distribution increases, the mean excess degree, 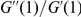, also increases. The number of two paths through a node of degree 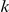 is 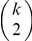. If we consider a node with mean excess degree 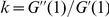, which is the mean degree of a new infected node early in the epidemic, the probability that two neighbors of that node are connected is
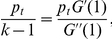
which will decrease as variance of the degree distribution decreases.

To measure the timescale of epidemics, we define 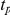 to be the time to peak incidence, 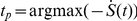. When we evaluated the influence of degree variance and clustering on the timescale of epidemics, we found that while clustering always slows the epidemic and increases 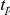, variance accelerates an epidemic and decreases 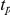 (Supporting Figure 2 in Text S1). We also found that 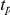 is much more elastic with respect to variance than 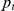 (Supporting Figure 2 in Text S1). The HK model is in close agreement with the clustering model (equations 13–17), but can differ by as much as 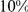 when 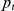 is large.

### The spread of infectious disease through households

Many respiratory diseases such as influenza spread through networks of close-proximity contacts. Transmission can be especially intense within households, where contacts are highly clustered. The clustering of close-proximity contacts that occurs within households is an important factor in the spread of such diseases and such clustering has been the subject of many mathematical models [16], [33]. In this section we illustrate how the model in equations 19–21 can be parameterized from real data that includes household contacts. The model developed below is designed for didactic purposes; it does not provide a realistic representation of a specific disease spreading in a specific population. This model excludes a number of complexities, such as age structure, clustering of non-household contacts, and dynamic partnerships. Nonetheless, the model illustrates the conditions under which it is important to include clustering of household contacts. Model misspecification can bias both model predictions and model-based estimates of parameter values.

To parameterize this model, we used data from the POLYMOD study [24], which consists of a sample of 7,290 individuals in eight European countries. These data are diary-based estimates of the number and type of contacts sufficient for transmission of a respiratory pathogen over a 24 hour period. Crucial for our purposes, the data provide a breakdown of contacts made both inside and outside of households. After pooling the data from each country, we find that the number of contacts outside of households was well described by a geometric distribution, which is generated by
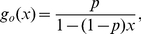
(31)with 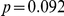. The geometric distribution was selected by the minimum AIC criterion in comparison with Poisson and negative binomial distributions fit to the data using maximum likelihood. For household sizes, we used the empirical distribution rather than fitting the data to an idealized distribution.To ensure that the system is computationally tractable, we limited the maximum household size at eight, and rounded down any households of larger size; only 2% of households included more than eight individuals. Letting the vector of dummy variables 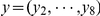 correspond to household sizes, the following generates the household size distribution:
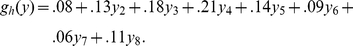
(32)The first term in 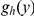 accounts for the probability of living alone. This model assumes that the household size is independent of the number of contacts made outside the household. This approximation is supported by the data, which shows very low correlation between the number of contacts reported within and outside of households (Pearson correlation coefficient 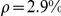). Consequently, the generating function for the entire system is the product of marginal PGFs.
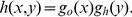
(33)For most respiratory diseases, it is reasonable to assume that the transmission rate within households, 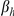, is greater than the transmission rate outside of households, 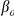 [34]. Applying the PGF 32 to the system of equations 19–21 and using the transmission rates 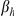 and 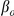 completes the model.

**Figure 4:**
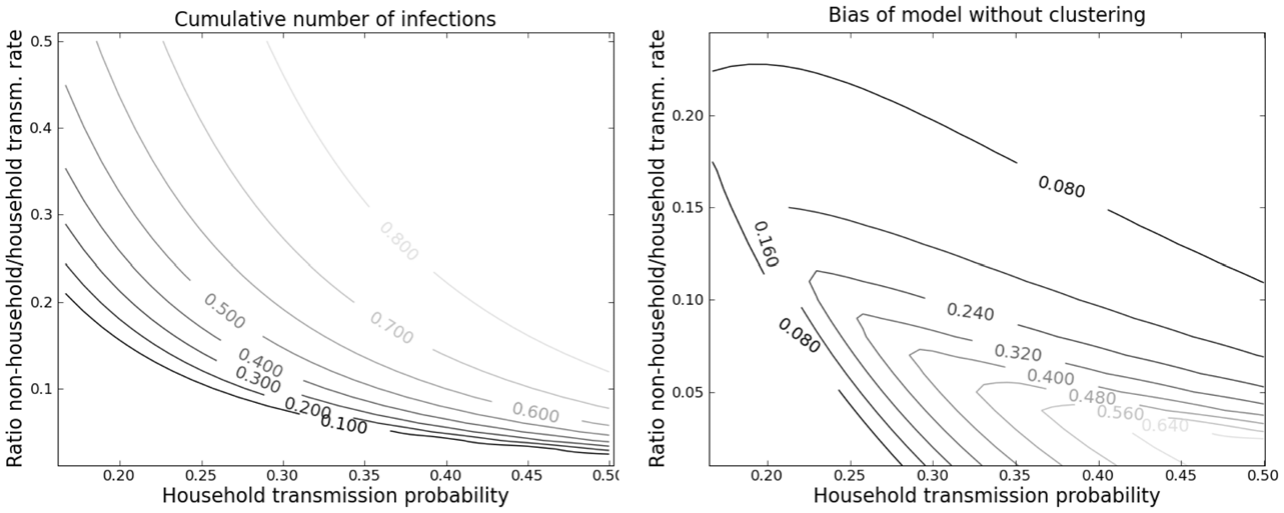
Epidemic size and bias of network models without clustering. Left: The final cumulative number of infections as predicted by the clique model is shown as a function of the transmissibility within households and the transmissibility for contacts outside of households. Right: The difference between final epidemic size in a model without clustering and the final size predicted by the clique model.

Figure 4 shows the final epidemic size (cumulative number of infections) for the clique model over a range of transmission probabilities both within and outside of households. The transmission probability is the per-edge probability that an infected will transmit prior to recovery, and is 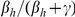 within households and 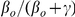 outside of households. The final size is much more sensitive to 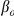 than 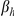 because the mean number of non-household contacts is much greater than household contacts (10.9 versus 3.3) and the household contacts only occur within cliques.

To determine the epidemiological significance of household clustering, we compared the clique model to a null model that had an identical degree distribution but no clustering. The null model retains household contacts with the transmission rate 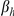, but in the null model, such edges do not appear in cliques. In general, the null model without clustering will over-estimate epidemic size. Consequently, null model-based estimates of the epidemiological importance of household contacts will tend to be inflated. The following discussion is oriented around the estimation of epidemic size given epidemic parameters. However, model misspecification will also bias estimates of transmission rates and other parameters made by fitting the network models to empirical epidemic data.

We have identified two sources of bias in the null model without clustering:

1. Clustering by household introduces redundancies relative to the null model that limit transmission, regardless of transmissibilities.
2. When there are two classes of edges (high transmissibility and low transmissibility), the household model aggregates the high transmissibility edges into the redundant parts of the network.

The second factor accounts for most of the bias in this example; clustering alone introduces little bias. For example, comparisons of the null model and clique model with 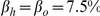 and 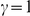 show that true final size is 29%, and the null model is biased by less than 0.36%.

The bias is greatest when transmissibility is high within households, but low outside of households (Figure 3). Outside of this small region, the null model can provide good approximation. Nevertheless, there is good reason to believe that for many real epidemics, the parameters will lie close to the region of high bias. For example, the per-day transmissibility of influenza within households has been estimated to be around 5% [34], and based on a 6 day infectious period, this implies a cumulative transmission probability of 20–30%. If transmission rates per edge outside of households are an order of magnitude less than household transmission rates, the network model without clustering may be biased by more than 25%.

## Discussion

We have investigated the interactive effects of clustering and the degree distribution of contact networks on the timescale and final size of infectious disease epidemics. For this purpose, we developed a model that generalizes the one presented in [17]. This model has previously been generalized in other dimensions [18], including the incorporation of simultaneous network dynamics, such as edge swapping [30], [35], populations with heterogeneous contact rates [36], multiple edge types with distinct transmission rates [28], preferential attachment [28], and growing networks with natural birth and mortality [37]. These extensions can be combined and extended further to model, for example, epidemics in clustered networks that also have dynamically rearranging ties, or networks in which larger clique sizes or other network motifs are prominent [15].

Model selection for epidemic dynamics in networks is a challenging problem; and our work has made two contributions to understanding the biases introduced by model misspecification. We have shown that when infectious periods vary among individuals, models that assume homogeneous transmissibility across all edges in a clustered network can be very biased; and the magnitude of this bias increases with the amount of clustering in the network. In contrast, bond percolation models that neglect variable infectious periods suffer negligible bias in configuration model networks without clustering [21].

The impact of clustering and degree distributions on SIR epidemic dynamics was previously investigated with the HK model [19]. We have compared that model to ours by calibrating the clustering coefficient of the HK model to match the fraction of links to triangles in ours. Our comparison indicates that the models are in close agreement when the variance of the degree distribution is high, but substantial differences in the expected final size and timescale of the epidemic exist when the degree distribution is homogeneous and clustering is extensive. This suggests that epidemic dynamics depend not only on the clustering coefficient, but also on the specific nature of clustering in the network. While the HK model is easy to parameterize when a population has a known clustering coefficient, our model facilitates parameterization using data with well defined cliques, such as human populations with household structure [16], [34].

This model allows the number of cliques of different sizes connected to a node to be correlated, but assumes that no two cliques connected to a node share other members. For example, it is not possible for two triangles connected to a node 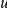 to share any nodes except for 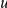. However, this feature of the model could be relaxed without much difficulty. A motif-based generalization of the configuration model was recently presented in [15] which provides one way of allowing triangles and other cliques to share more than one node.

Contact data increasingly provide the information necessary to parameterize network models including the one presented here. Social network studies often ascertain degree distributions and clustering coefficients [38], [39] and epidemiological surveillance data often provide partnership durations and measures of concurrency [28], [40]. We have demonstrated how such data can be used to parameterize the network structure parameters of our model, with a focus on the clustering introduced by household structure, and we have shown the value of explicitly considering this component of human contact patterns in epidemiological models. Without it, models may overestimate both the epidemiological risk of a population and the extent to which household contact contribute to that risk.

## Supporting Information

Text S1Supporting information. This supplement contains supporting Figures and methods. including a bond percolation solution for final epidemic size in models with generalized distributions of clique sizes, and a generalization of the 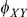 system to clique sizes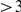.(PDF)
