# Correction: Effects of Heterogeneous and Clustered Contact Patterns on Infectious Disease Dynamics

**DOI:** 10.1371/annotation/85b99614-44b4-4052-9195-a77d52dbdc05

**Published:** 2011-07-05

**Authors:** Erik M. Volz, Joel C. Miller, Alison Galvani, Lauren Ancel Meyers

The funding section is incomplete. The complete funding information is: "The authors acknowledge financial support from NIH U01 GM087719. JCM acknowledges support from the RAPIDD program of Department of Homeland Security and the NIH Fogarty International Center; and the United States National Institutes of Health Models of Infectious Disease Agent Study program through cooperative agreement 1U54GM088558. The funders had no role in study design, data collection and analysis, decision to publish, or preparation of the manuscript."

