# Supplementary material for: Effects of Heterogeneous and Clustered Contact Patterns on Infectious Disease Dynamics
Source: PLoS Comput Biol. 2011 Jun 2;7(6):e1002042. doi: 10.1371/journal.pcbi.1002042 (PMC3107246; doi:10.1371/journal.pcbi.1002042)
Supplement: Text S1 — Supporting information. This supplement contains supporting Figures and methods. including a bond percolation solution for final epidemic size in models with generalized distributions of clique sizes, and a generalization of the system to clique sizes. (PDF) [file pcbi.1002042.s001.pdf]

Supplementary Text S1

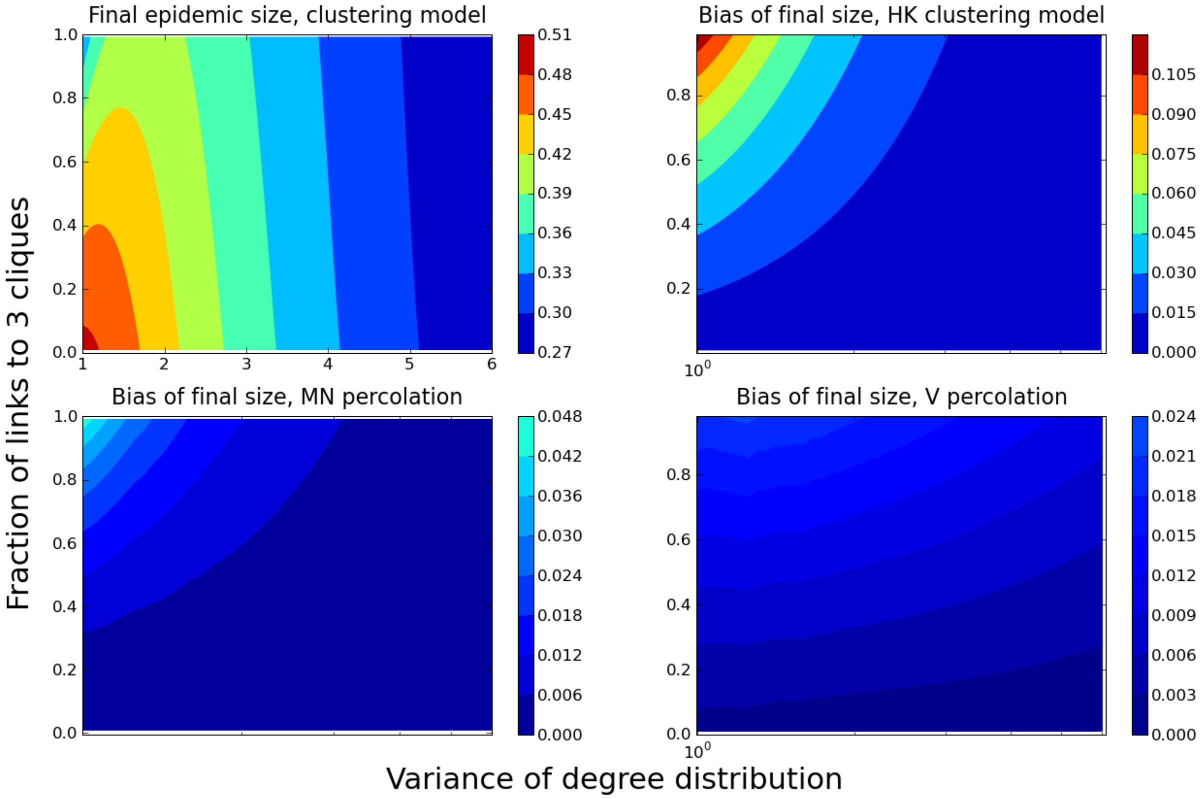

Figure 1. Joint impact of clustering and variance of the degree distribution on epidemic size. Upper left: The final epidemic size is shown as a function of clustering (the fraction of edges in triangles) and the variance of the degree distribution. All results are based on a solution of equations 13–17 in the main text . $\beta = \gamma = 1$. The remaining panels show the absolute value of the difference between final size predicted by the clustering model (equations 13–17 in the main text) and final size predicted by alternative models.The heatmaps were calibrated to have the same color scale.

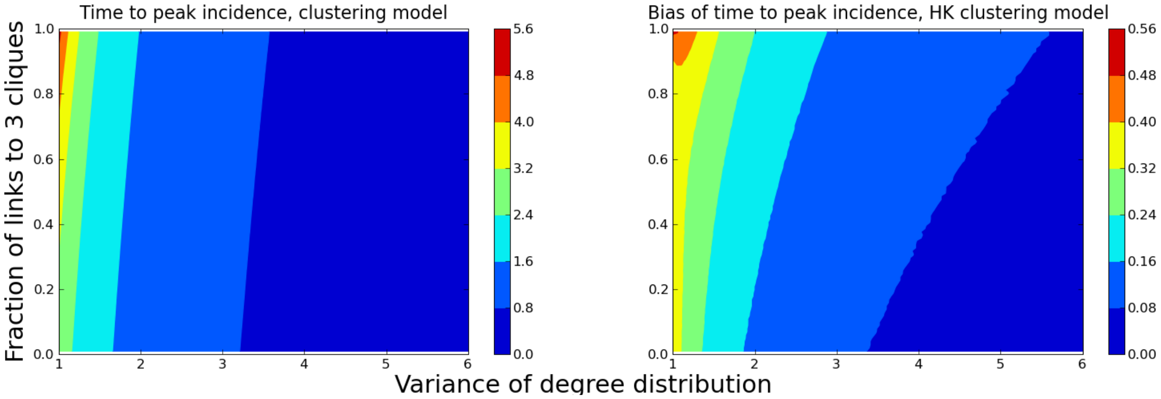

Figure 2. Joint impact of clustering and variance of the degree distribution on timing of epidemic peak. Left: The time to peak epidemic incidence as a function of the fraction of edges in triangles, p_t, and the variance of the degree distribution. Results are based on a solution to equations 13–17 in the main text, with $\beta = \gamma = 1$ and a degree distribution generate by equation 30 in the main text. Right: The absolute value of the difference between the time of peak incidence predicted by the HK model and the clustering model (equations 13–17 in the main text).

Bond percolation solution for final epidemic size in models with generalized distributions of clique sizes

Consider a clique of t nodes. Assume that n of the nodes would receive infection from outside the clique. For the purposes of calculating the probability that a given node in that clique ultimately gets infected, it makes no difference what order the infections are introduced. In fact, we can assume that all n of those nodes are infected at the same time. This is the assumption we will make.

Define $\alpha_t(n)$ to be the probability that a node u is not infected by any node in a clique of size t of which u is a member; furthermore we assume u is not among the n introduced infections. A few cases are trivial: $\alpha_t(0) = 0$: if there is no introduced infection, it cannot become infected. Similarly, if all other nodes are infected, the probability that each does not lead to infection of u is independent, with probability $\gamma/(\beta+\gamma)$ so $\alpha_t(t-1) = [\beta/(\beta+\gamma)]^{t-1}$.

Now consider the general case for $\alpha_t(n)$. Infection occurs at rate $\beta n(t-n)$ and recovery at rate γn. The probability the first event is an infection is $\beta n(t-n)/[\beta n(t-n)+\gamma n] = \beta(t-n)/[\beta(t-n)+\gamma]$. If the first event is an infection, the probability it is not an infection of u is $(t-n-1)/(t-n)$. Assuming this happens, the probability u is ultimately infected is the same as if there were $n+1$ introductions, *i.e.,* $\alpha_t(n+1)$. The probability the first event is a recovery is $\gamma/[\beta(t-n)+\gamma]$. Assuming that this happens, the recovered node no longer plays a role: the probability u is eventually infected is the same as in a clique of one fewer node with one fewer introduced infection, $\alpha_{t-1}(n-1)$. This leads to the system

$$\alpha_t(n) = \frac{\beta(t-n-1)}{\gamma+\beta(t-n)}\alpha_t(n+1) + \frac{\gamma}{\gamma+\beta(t-n)}\alpha_{t-1}(n-1)$$

with boundary conditions

$$\alpha_t(0) = 1 \qquad \alpha_t(t-1) = \left(\frac{\gamma}{\gamma+\beta}\right)^{t-1}$$

This is a straightforward system to solve numerically.

Then $q_t = g^{(x_t)}(\theta_2, \theta_3, \ldots)/g^{(x_t)}(1, 1, \ldots)$ and

$$\theta_t = \sum_{n=0}^{t-1} \binom{t-1}{n} q_t^n (1-q_t)^{n-1} \alpha_t(n)$$

This can be solved iteratively starting with the assumption that $q_t = 0$

Generalization of the ϕ_{XY} system to clique sizes > 3

The system of equations based on probabilities ϕ can also be extended to cliques of size > 3 as follows. Consider a clique of size k containing the test node u, let $\phi(n_s, n_I, n_R)$ denote the probability that there are $n_S \geq 0$ susceptible, $n_I \geq 0$ infected, and $n_R \geq 0$ recovered neighbors in the clique ($n_S + n_I + n_R = k-1$). Let θ_k be the probability that no node in the k-clique has transmitted to u.

Assuming $n_S > 0$ (there are susceptible nodes) there is flux from $\phi(n_s, n_I, n_R)$ to $\phi((n_S-1), (n_I+1), n_R)$ resulting from infection of a susceptible nighbor. This occurs at rate $(A_k + n_I\beta)n_S$ where A_k is the rate at which a neighbor in a k-clique is infected by a node outside the clique. If $n_I > 0$, then there is also flux from $\phi(n_s, n_I, n_R)$ to $\phi(n_S, (n_I-1), (n_R+1))$ resulting from recovery of an infected neighbor. This occurs at rate γn_I. The only remaining flux is from $\phi(n_s, n_I, n_R)$ to $1-\theta_k$ representing infection of u by a neighbor. This occurs at rate βn_I.

To calculate A_k, we must find the flux from $\phi((k-1), 0, 0)$ to $\phi((k-2), 1, 0)$ which occurs at rate $(k-1)A_k\phi((k-1), 0, 0)$. To accomplish this, we explicitly find $\phi((k-1), 0, 0)$ and differentiate it. Then we find $A_k = -\dot{\phi}((k-1), 0, 0)/(k-1)\phi((k-1), 0, 0)$ where

$$\phi((k-1), 0, 0) = \left(\frac{g^{(y_k)}(\theta)}{g^{(y_k)}(1)}\right)^{k-1}.$$
